# 1,5-Bis[(*E*)-1-(2-hydroxy­phen­yl)ethyl­idene]carbonohydrazide dimethyl sulfoxide solvate

**DOI:** 10.1107/S1600536809044985

**Published:** 2009-10-31

**Authors:** Julio Zukerman-Schpector, Md. Abu Affan, Siong Wan Foo, Edward R. T. Tiekink

**Affiliations:** a Department of Chemistry, Universidade Federal de São Carlos, 13565-905 São Carlos SP, Brazil; b Department of Chemistry, Faculty of Resource Science and Technology, Universiti Malaysia Sarawak, 94300 Kota Samarahan, Sarawak, Malaysia; c Department of Chemistry, University of Malaya, 50603 Kuala Lumpur, Malaysia

## Abstract

The title dimethyl sulfoxide (DMSO) solvate, C_17_H_18_N_4_O_3_·C_2_H_6_OS, shows the disubstituted urea derivative to adopt an almost planar geometry (r.m.s. deviation for non-H atoms = 0.132 Å); the mol­ecule has non-crystallographic twofold mol­ecular symmetry. This conformation is stabilized by two intra­molecular O—H⋯N hydrogen bonds. The components of the crystal are connected by N—H⋯O hydrogen bonds, whereby both amine H atoms are connected to a DMSO O atom, and C—H⋯O contacts involving the DMSO H and urea carbonyl atoms, forming a supra­molecular chain along the *c* axis. The chains associate *via* C—H⋯π inter­actions.

## Related literature

For background and recent studies on the biological activity of tin/organotin compounds, see: Gielen & Tiekink (2005 ▶); Affan *et al.* (2009 ▶).
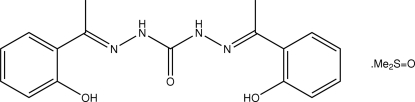


## Experimental

### 

#### Crystal data

C_17_H_18_N_4_O_3_·C_2_H_6_OS
*M* *_r_* = 404.48
Monoclinic, 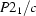

*a* = 15.3260 (19) Å
*b* = 7.1248 (7) Å
*c* = 18.439 (2) Å
β = 102.724 (2)°
*V* = 1964.0 (4) Å^3^
*Z* = 4
Mo *K*α radiation
μ = 0.20 mm^−1^
*T* = 153 K
0.32 × 0.30 × 0.15 mm

#### Data collection

Rigaku Saturn724 diffractometer
Absorption correction: multi-scan (*ABSCOR*; Higashi, 1995 ▶) *T*_min_ = 0.661, *T*_max_ = 1.000
21771 measured reflections
4493 independent reflections
4369 reflections with *I* > 2σ(*I*)
*R*_int_ = 0.030

#### Refinement

*R*[*F* ^2^ > 2σ(*F* ^2^)] = 0.041
*wR*(*F* ^2^) = 0.110
*S* = 1.08
4493 reflections
269 parameters
4 restraints
H-atom parameters constrained
Δρ_max_ = 0.32 e Å^−3^
Δρ_min_ = −0.32 e Å^−3^

### 

Data collection: *CrystalClear* (Rigaku/MSC, 2005 ▶); cell refinement: *CrystalClear*; data reduction: *CrystalClear*; program(s) used to solve structure: *SIR97* (Altomare *et al.*, 1999 ▶); program(s) used to refine structure: *SHELXL97* (Sheldrick, 2008 ▶); molecular graphics: *DIAMOND* (Brandenburg, 2006 ▶); software used to prepare material for publication: *SHELXL97*.

## Supplementary Material

Crystal structure: contains datablocks general, I. DOI: 10.1107/S1600536809044985/hb5198sup1.cif

Structure factors: contains datablocks I. DOI: 10.1107/S1600536809044985/hb5198Isup2.hkl

Additional supplementary materials:  crystallographic information; 3D view; checkCIF report

## Figures and Tables

**Table 1:** Hydrogen-bond geometry (Å, °)

*D*—H⋯*A*	*D*—H	H⋯*A*	*D*⋯*A*	*D*—H⋯*A*
O1—H1*o*⋯N1	0.84	1.79	2.5682 (15)	153
O3—H3*o*⋯N4	0.84	1.78	2.5450 (15)	150
N2—H2*n*⋯O4^i^	0.88	1.94	2.7674 (15)	156
N3—H3*n*⋯O4^i^	0.88	1.97	2.7907 (15)	154
C19—H19*b*⋯O2^ii^	0.98	2.49	3.2167 (17)	131
C8—H8*A*⋯*Cg*2^iii^	0.98	2.83	3.5018 (16)	127

Symmetry codes: (i) 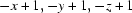; (ii) 
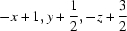
; (iii) 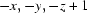. *Cg*2 is the centroid of the C12–C17 ring.

**Fig. 1.**
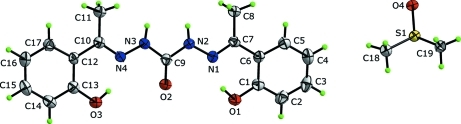
Molecular structures of the molecules comprising the asymmetric unit in (I) showing atom-labelling scheme and displacement ellipsoids at the 70% probability level.

**Fig. 2.**
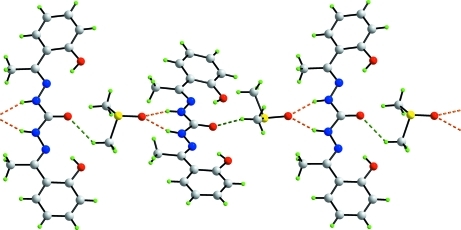
Supramolecular chain formation along the c axis in (I) mediated by N–H···O (orange dashed lines) hydrogen bonds and C–H···O (green dashed lines) contacts.

**Fig. 3.**
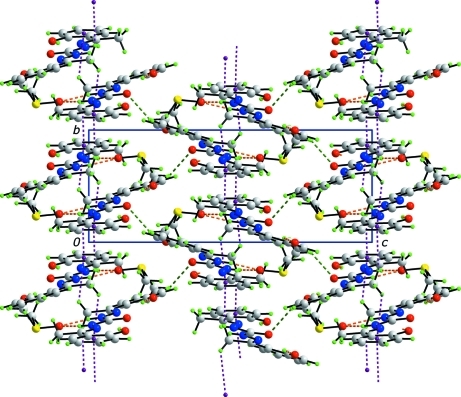
View in projection down the a axis of the crystal packing in (I), highlighting the C–H···π interactions (purple dashed lines). The N–H···O (orange dashed lines) hydrogen bonds and C–H···O (green dashed lines) contacts are also shown.

## supplementary crystallographic information

### Comment

The title compound, (I), was prepared as a part of on-going studies into the biological activity of organotin compounds (Gielen & Tiekink, 2005; Affan *et al.*, 2009). Crystals of (I) comprise equal quantities of a disubstituted urea molecule and a solvent dimethyl sulfoxide molecule, Fig. 1. The urea derivative, which has molecular twofold symmetry (non-crystallographic), is essentially planar as seen in the r.m.s. value of 0.132 Å for all non-H atoms. The arrangement is stabilized by two internal O–H···N hydrogen bonds, Table 1.

In the crystal structure, the two amine-H atoms form hydrogen bonds to the DMSO-O atom to generate a supramolecular dimer, Table 1. The dimers thus formed are connected into a supramolecular chain along the *c* axis *via* C–H···O contacts involving the carbonyl-O4 atoms and DMSO-H atoms, Table 1 and Fig. 2. The chains are connected by C–H···π contacts to consolidate the crystal structure, Table 1 and Fig. 3.

### Experimental

Carbohydrazide (0.90 g, 10 mmol) and 2-hydroxyacetophenone (2.72 g, 20 mmol) in dry methanol (40 ml) were heated at reflux for 4 h and cooled to ambient temperature. During cooling process, white microcrystals formed and were filtered off. The microcrystals, (I), were washed several times with small amounts of cold methanol and cold hexane. Crude (I) was recrystallized from methanol and dried *in vacuo* over silica gel. Yield: 1.99 g, 55%; m. pt. 467–468 K. Analysis. Calculated for C_17_H_18_N_4_O_3_: C, 62.56; H, 5.56; N, 17.17%. Found: C, 62.28; H, 5.61; N, 17.02%. UV-visible (DMSO) λ_max_: 282, 317, 382 nm. FT—IR (KBr disc) ν: 3453 (m, OH), 3346 (m, NH), 1701 (s, CONH), 1615 (s, C=N), 1000 (w, N—N) cm^-1. 1^H NMR (DMSO-d6) δ: 10.08 (s, 1H, OH), 8.30 (s, br, 1H, CONH), 7.78–7.76 (d, 1H, phenyl C3—H), 7.56–7.55 (d, 1H, phenyl C6—H), 7.27–7.24 (t, 1H, phenyl C4—H), 6.90–6.86 (t, 1H, phenyl C5—H), 2.31 (s, 3H, N=C—CH_3_) p.p.m. ^13^C NMR (CDCl_3_) δ: 168.19 (1 C, HN—C=O), 158.04 (2 C, C=N), 155.59, 151.98, 130.71, 128.06, 118.77, 117.09 (12 C, benzene ring), 13.16 (2 C, CH_3_) p.p.m. Crystals for the diffraction study were obtained from a dimethyl sulfoxide solution of (I).

### Refinement

Carbon-bound H-atoms were placed in calculated positions (C–H 0.95–0.98 Å) and were included in the refinement in the riding model approximation with *U*_iso_(H) set to 1.2–1.5*U*_eq_(C). The O– and N-bound H-atoms were located in a difference Fourier map and were refined with O–H and N–H restraints of 0.840±0.001 Å and 0.880±0.001 Å, respectively, and with *U*_iso_(H) = 1.2*U*_eq_(N) and 1.5*U*_eq_(O).

### Crystal data

**Table:**

C_17_H_18_N_4_O_3_·C_2_H_6_OS	*F*(000) = 856
*M**_r_* = 404.48	*D*_x_ = 1.368 Mg m^−^^3^
Monoclinic, *P*2_1_/*c*	Mo *K*α radiation, λ = 0.71073 Å
Hall symbol: -P 2ybc	Cell parameters from 6256 reflections
*a* = 15.3260 (19) Å	θ = 2.7–30.3°
*b* = 7.1248 (7) Å	µ = 0.20 mm^−^^1^
*c* = 18.439 (2) Å	*T* = 153 K
β = 102.724 (2)°	Prism, colourless
*V* = 1964.0 (4) Å^3^	0.32 × 0.30 × 0.15 mm
*Z* = 4	

### Data collection

**Table:**

Rigaku Saturn724 diffractometer	4493 independent reflections
Radiation source: sealed tube	4369 reflections with *I* > 2σ(*I*)
graphite	*R*_int_ = 0.030
Detector resolution: 28.5714 pixels mm^-1^	θ_max_ = 27.5°, θ_min_ = 2.3°
ω scans	*h* = −19→18
Absorption correction: multi-scan (*ABSCOR*; Higashi, 1995)	*k* = −9→9
*T*_min_ = 0.661, *T*_max_ = 1.000	*l* = −23→23
21771 measured reflections	

### Refinement

**Table:**

Refinement on *F*^2^	Primary atom site location: structure-invariant direct methods
Least-squares matrix: full	Secondary atom site location: difference Fourier map
*R*[*F*^2^ > 2σ(*F*^2^)] = 0.041	Hydrogen site location: inferred from neighbouring sites
*wR*(*F*^2^) = 0.110	H-atom parameters constrained
*S* = 1.08	*w* = 1/[σ^2^(*F*_o_^2^) + (0.0572*P*)^2^ + 0.8148*P*] where *P* = (*F*_o_^2^ + 2*F*_c_^2^)/3
4493 reflections	(Δ/σ)_max_ = 0.001
269 parameters	Δρ_max_ = 0.32 e Å^−^^3^
4 restraints	Δρ_min_ = −0.32 e Å^−^^3^

### Special details

**Table:**

Geometry. All s.u.'s (except the s.u. in the dihedral angle between two l.s. planes) are estimated using the full covariance matrix. The cell s.u.'s are taken into account individually in the estimation of s.u.'s in distances, angles and torsion angles; correlations between s.u.'s in cell parameters are only used when they are defined by crystal symmetry. An approximate (isotropic) treatment of cell s.u.'s is used for estimating s.u.'s involving l.s. planes.
Refinement. Refinement of *F*^2^ against ALL reflections. The weighted *R*-factor *wR* and goodness of fit *S* are based on *F*^2^, conventional *R*-factors *R* are based on *F*, with *F* set to zero for negative *F*^2^. The threshold expression of *F*^2^ > 2σ(*F*^2^) is used only for calculating *R*-factors(gt) *etc*. and is not relevant to the choice of reflections for refinement. *R*-factors based on *F*^2^ are statistically about twice as large as those based on *F*, and *R*- factors based on ALL data will be even larger.

### Fractional atomic coordinates and isotropic or equivalent isotropic displacement parameters (Å^2^)

**Table:**

	*x*	*y*	*z*	*U*_iso_*/*U*_eq_	
O1	0.31503 (7)	−0.00388 (17)	0.72741 (5)	0.0315 (2)	
H1O	0.2776	0.0352	0.6901	0.047*	
O2	0.08573 (6)	0.17694 (15)	0.63438 (5)	0.0258 (2)	
O3	−0.14836 (6)	0.29124 (14)	0.62863 (5)	0.0248 (2)	
H3O	−0.1046	0.27566	0.6087	0.037*	
N1	0.24627 (7)	0.12659 (16)	0.59756 (6)	0.0207 (2)	
N2	0.17027 (7)	0.17151 (17)	0.54592 (6)	0.0221 (2)	
H2N	0.1683	0.1867	0.4982	0.026*	
N3	0.02270 (7)	0.24035 (16)	0.51181 (6)	0.0221 (2)	
H3N	0.0339	0.2475	0.4671	0.027*	
N4	−0.05924 (7)	0.26766 (15)	0.52770 (6)	0.0204 (2)	
C1	0.39368 (9)	0.00078 (19)	0.70500 (8)	0.0233 (3)	
C2	0.47033 (10)	−0.0498 (2)	0.75744 (8)	0.0289 (3)	
H2	0.4655	−0.0855	0.8060	0.035*	
C3	0.55316 (10)	−0.0486 (2)	0.73960 (9)	0.0306 (3)	
H3	0.6050	−0.0826	0.7759	0.037*	
C4	0.56065 (9)	0.0023 (2)	0.66867 (9)	0.0304 (3)	
H4	0.6176	0.0036	0.6562	0.037*	
C5	0.48487 (9)	0.0513 (2)	0.61617 (8)	0.0259 (3)	
H5	0.4907	0.0848	0.5676	0.031*	
C6	0.39962 (8)	0.05313 (17)	0.63249 (7)	0.0200 (2)	
C7	0.31992 (8)	0.10518 (17)	0.57510 (7)	0.0195 (2)	
C8	0.32461 (9)	0.1268 (2)	0.49519 (7)	0.0256 (3)	
H8A	0.2859	0.0332	0.4651	0.038*	
H8B	0.3864	0.1082	0.4903	0.038*	
H8C	0.3046	0.2530	0.4780	0.038*	
C9	0.09245 (8)	0.19450 (18)	0.56997 (7)	0.0202 (3)	
C10	−0.12715 (8)	0.30637 (18)	0.47447 (7)	0.0199 (3)	
C11	−0.12059 (9)	0.3222 (2)	0.39461 (7)	0.0278 (3)	
H11A	−0.0659	0.3903	0.3917	0.042*	
H11B	−0.1727	0.3904	0.3665	0.042*	
H11C	−0.1189	0.1964	0.3735	0.042*	
C12	−0.21323 (8)	0.33360 (17)	0.49676 (7)	0.0193 (2)	
C13	−0.21935 (9)	0.32848 (18)	0.57248 (7)	0.0213 (3)	
C14	−0.30117 (9)	0.36037 (19)	0.59185 (8)	0.0253 (3)	
H14	−0.3042	0.3618	0.6428	0.030*	
C15	−0.37788 (9)	0.3899 (2)	0.53742 (9)	0.0282 (3)	
H15	−0.4333	0.4109	0.5512	0.034*	
C16	−0.37441 (9)	0.3891 (2)	0.46264 (8)	0.0277 (3)	
H16	−0.4274	0.4061	0.4253	0.033*	
C17	−0.29285 (9)	0.36309 (19)	0.44331 (8)	0.0240 (3)	
H17	−0.2907	0.3653	0.3922	0.029*	
S1	0.88984 (2)	0.73077 (5)	0.686182 (17)	0.02070 (11)	
O4	0.88705 (7)	0.75110 (16)	0.60411 (5)	0.0286 (2)	
C18	0.79058 (9)	0.8403 (2)	0.70127 (8)	0.0286 (3)	
H18A	0.7859	0.9673	0.6804	0.043*	
H18B	0.7930	0.8470	0.7548	0.043*	
H18C	0.7383	0.7666	0.6769	0.043*	
C19	0.96707 (9)	0.9044 (2)	0.73080 (7)	0.0258 (3)	
H19A	1.0273	0.8722	0.7250	0.039*	
H19B	0.9664	0.9093	0.7838	0.039*	
H19C	0.9499	1.0271	0.7081	0.039*	

### Atomic displacement parameters (Å^2^)

**Table:**

	*U*^11^	*U*^22^	*U*^33^	*U*^12^	*U*^13^	*U*^23^
O1	0.0242 (5)	0.0477 (6)	0.0232 (5)	0.0023 (5)	0.0068 (4)	0.0100 (4)
O2	0.0235 (5)	0.0351 (5)	0.0197 (4)	0.0028 (4)	0.0067 (4)	0.0018 (4)
O3	0.0235 (5)	0.0322 (5)	0.0190 (5)	0.0008 (4)	0.0053 (4)	0.0004 (4)
N1	0.0187 (5)	0.0233 (5)	0.0197 (5)	0.0011 (4)	0.0030 (4)	0.0000 (4)
N2	0.0183 (5)	0.0312 (6)	0.0165 (5)	0.0013 (4)	0.0033 (4)	0.0017 (4)
N3	0.0179 (5)	0.0305 (6)	0.0190 (5)	0.0015 (4)	0.0062 (4)	0.0013 (4)
N4	0.0177 (5)	0.0226 (5)	0.0219 (5)	0.0000 (4)	0.0065 (4)	−0.0004 (4)
C1	0.0229 (6)	0.0222 (6)	0.0247 (6)	−0.0002 (5)	0.0051 (5)	0.0021 (5)
C2	0.0289 (7)	0.0282 (7)	0.0276 (7)	−0.0007 (6)	0.0017 (5)	0.0078 (6)
C3	0.0241 (7)	0.0268 (7)	0.0367 (8)	0.0028 (6)	−0.0026 (6)	0.0054 (6)
C4	0.0204 (6)	0.0315 (7)	0.0392 (8)	0.0020 (6)	0.0062 (6)	0.0000 (6)
C5	0.0224 (6)	0.0287 (7)	0.0272 (7)	−0.0001 (5)	0.0065 (5)	−0.0016 (5)
C6	0.0193 (6)	0.0188 (6)	0.0216 (6)	−0.0002 (5)	0.0039 (5)	−0.0014 (5)
C7	0.0210 (6)	0.0190 (6)	0.0191 (6)	−0.0007 (5)	0.0058 (5)	−0.0016 (4)
C8	0.0241 (6)	0.0342 (7)	0.0194 (6)	0.0016 (5)	0.0064 (5)	0.0000 (5)
C9	0.0205 (6)	0.0200 (6)	0.0204 (6)	−0.0010 (5)	0.0049 (5)	−0.0004 (5)
C10	0.0212 (6)	0.0183 (6)	0.0204 (6)	−0.0014 (5)	0.0054 (5)	−0.0007 (5)
C11	0.0242 (6)	0.0390 (8)	0.0210 (6)	0.0024 (6)	0.0067 (5)	0.0027 (6)
C12	0.0190 (6)	0.0174 (5)	0.0217 (6)	−0.0012 (5)	0.0051 (5)	−0.0010 (5)
C13	0.0225 (6)	0.0182 (6)	0.0238 (6)	−0.0022 (5)	0.0062 (5)	−0.0011 (5)
C14	0.0277 (7)	0.0238 (6)	0.0276 (6)	−0.0011 (5)	0.0129 (5)	−0.0018 (5)
C15	0.0213 (6)	0.0269 (7)	0.0395 (8)	0.0004 (5)	0.0130 (6)	−0.0019 (6)
C16	0.0198 (6)	0.0289 (7)	0.0328 (7)	0.0016 (5)	0.0024 (5)	0.0000 (6)
C17	0.0232 (6)	0.0242 (6)	0.0242 (6)	−0.0003 (5)	0.0042 (5)	−0.0012 (5)
S1	0.01945 (17)	0.02472 (18)	0.01720 (17)	0.00048 (11)	0.00244 (12)	−0.00063 (11)
O4	0.0223 (5)	0.0470 (6)	0.0162 (5)	0.0010 (4)	0.0034 (4)	−0.0024 (4)
C18	0.0214 (6)	0.0409 (8)	0.0241 (6)	0.0025 (6)	0.0060 (5)	−0.0001 (6)
C19	0.0239 (6)	0.0318 (7)	0.0206 (6)	−0.0056 (5)	0.0027 (5)	−0.0006 (5)

### Geometric parameters (Å, °)

**Table:**

O1—C1	1.3580 (16)	C8—H8B	0.9800
O1—H1O	0.8401	C8—H8C	0.9800
O2—C9	1.2210 (16)	C10—C12	1.4785 (17)
O3—C13	1.3528 (16)	C10—C11	1.5021 (17)
O3—H3O	0.8400	C11—H11A	0.9800
N1—C7	1.2942 (16)	C11—H11B	0.9800
N1—N2	1.3700 (15)	C11—H11C	0.9800
N2—C9	1.3707 (16)	C12—C17	1.4053 (18)
N2—H2N	0.8799	C12—C13	1.4202 (17)
N3—N4	1.3648 (15)	C13—C14	1.3961 (18)
N3—C9	1.3764 (17)	C14—C15	1.383 (2)
N3—H3N	0.8799	C14—H14	0.9500
N4—C10	1.2933 (17)	C15—C16	1.392 (2)
C1—C2	1.3944 (19)	C15—H15	0.9500
C1—C6	1.4103 (18)	C16—C17	1.3856 (19)
C2—C3	1.380 (2)	C16—H16	0.9500
C2—H2	0.9500	C17—H17	0.9500
C3—C4	1.386 (2)	S1—O4	1.5115 (10)
C3—H3	0.9500	S1—C19	1.7830 (14)
C4—C5	1.383 (2)	S1—C18	1.7852 (14)
C4—H4	0.9500	C18—H18A	0.9800
C5—C6	1.4039 (18)	C18—H18B	0.9800
C5—H5	0.9500	C18—H18C	0.9800
C6—C7	1.4765 (17)	C19—H19A	0.9800
C7—C8	1.4987 (17)	C19—H19B	0.9800
C8—H8A	0.9800	C19—H19C	0.9800
			
C1—O1—H1O	103.5	N4—C10—C11	122.93 (11)
C13—O3—H3O	106.0	C12—C10—C11	121.27 (11)
C7—N1—N2	118.23 (11)	C10—C11—H11A	109.5
N1—N2—C9	118.05 (10)	C10—C11—H11B	109.5
N1—N2—H2N	124.1	H11A—C11—H11B	109.5
C9—N2—H2N	117.9	C10—C11—H11C	109.5
N4—N3—C9	117.39 (11)	H11A—C11—H11C	109.5
N4—N3—H3N	124.8	H11B—C11—H11C	109.5
C9—N3—H3N	117.7	C17—C12—C13	117.26 (11)
C10—N4—N3	119.49 (11)	C17—C12—C10	120.98 (11)
O1—C1—C2	116.83 (12)	C13—C12—C10	121.75 (11)
O1—C1—C6	122.81 (11)	O3—C13—C14	116.90 (12)
C2—C1—C6	120.35 (12)	O3—C13—C12	122.77 (11)
C3—C2—C1	120.79 (13)	C14—C13—C12	120.33 (12)
C3—C2—H2	119.6	C15—C14—C13	120.45 (13)
C1—C2—H2	119.6	C15—C14—H14	119.8
C2—C3—C4	119.89 (13)	C13—C14—H14	119.8
C2—C3—H3	120.1	C14—C15—C16	120.42 (12)
C4—C3—H3	120.1	C14—C15—H15	119.8
C5—C4—C3	119.68 (13)	C16—C15—H15	119.8
C5—C4—H4	120.2	C17—C16—C15	119.28 (13)
C3—C4—H4	120.2	C17—C16—H16	120.4
C4—C5—C6	122.01 (13)	C15—C16—H16	120.4
C4—C5—H5	119.0	C16—C17—C12	122.19 (12)
C6—C5—H5	119.0	C16—C17—H17	118.9
C5—C6—C1	117.27 (12)	C12—C17—H17	118.9
C5—C6—C7	120.72 (12)	O4—S1—C19	105.37 (6)
C1—C6—C7	122.01 (11)	O4—S1—C18	106.08 (6)
N1—C7—C6	116.26 (11)	C19—S1—C18	97.28 (7)
N1—C7—C8	122.45 (11)	S1—C18—H18A	109.5
C6—C7—C8	121.28 (11)	S1—C18—H18B	109.5
C7—C8—H8A	109.5	H18A—C18—H18B	109.5
C7—C8—H8B	109.5	S1—C18—H18C	109.5
H8A—C8—H8B	109.5	H18A—C18—H18C	109.5
C7—C8—H8C	109.5	H18B—C18—H18C	109.5
H8A—C8—H8C	109.5	S1—C19—H19A	109.5
H8B—C8—H8C	109.5	S1—C19—H19B	109.5
O2—C9—N2	124.62 (12)	H19A—C19—H19B	109.5
O2—C9—N3	124.39 (12)	S1—C19—H19C	109.5
N2—C9—N3	110.99 (11)	H19A—C19—H19C	109.5
N4—C10—C12	115.80 (11)	H19B—C19—H19C	109.5
			
C7—N1—N2—C9	−179.85 (12)	N1—N2—C9—N3	179.57 (11)
C9—N3—N4—C10	−178.29 (11)	N4—N3—C9—O2	0.0 (2)
O1—C1—C2—C3	−179.82 (13)	N4—N3—C9—N2	−179.96 (11)
C6—C1—C2—C3	0.3 (2)	N3—N4—C10—C12	179.97 (11)
C1—C2—C3—C4	−0.4 (2)	N3—N4—C10—C11	0.31 (19)
C2—C3—C4—C5	−0.1 (2)	N4—C10—C12—C17	−175.46 (12)
C3—C4—C5—C6	0.6 (2)	C11—C10—C12—C17	4.20 (19)
C4—C5—C6—C1	−0.6 (2)	N4—C10—C12—C13	3.63 (18)
C4—C5—C6—C7	−179.69 (13)	C11—C10—C12—C13	−176.71 (12)
O1—C1—C6—C5	−179.69 (13)	C17—C12—C13—O3	176.40 (12)
C2—C1—C6—C5	0.1 (2)	C10—C12—C13—O3	−2.72 (19)
O1—C1—C6—C7	−0.6 (2)	C17—C12—C13—C14	−2.83 (18)
C2—C1—C6—C7	179.18 (12)	C10—C12—C13—C14	178.05 (12)
N2—N1—C7—C6	−178.82 (11)	O3—C13—C14—C15	−176.70 (12)
N2—N1—C7—C8	−0.32 (19)	C12—C13—C14—C15	2.6 (2)
C5—C6—C7—N1	−171.33 (12)	C13—C14—C15—C16	−0.3 (2)
C1—C6—C7—N1	9.66 (18)	C14—C15—C16—C17	−1.7 (2)
C5—C6—C7—C8	10.15 (19)	C15—C16—C17—C12	1.3 (2)
C1—C6—C7—C8	−168.86 (12)	C13—C12—C17—C16	0.9 (2)
N1—N2—C9—O2	−0.4 (2)	C10—C12—C17—C16	−179.98 (12)

### Hydrogen-bond geometry (Å, °)

**Table:**

*D*—H···*A*	*D*—H	H···*A*	*D*···*A*	*D*—H···*A*
O1—H1o···N1	0.84	1.79	2.5682 (15)	153
O3—H3o···N4	0.84	1.78	2.5450 (15)	150
N2—H2n···O4^i^	0.88	1.94	2.7674 (15)	156
N3—H3n···O4^i^	0.88	1.97	2.7907 (15)	154
C19—H19b···O2^ii^	0.98	2.49	3.2167 (17)	131
C8—H8A···Cg2^iii^	0.98	2.83	3.5018 (16)	127

Symmetry codes:  (i) −*x*+1, −*y*+1, −*z*+1; (ii) −*x*+1, *y*+1/2, −*z*+3/2; (iii) −*x*, −*y*, −*z*+1.
